# Biological effective dose in analysis of rectal dose in prostate cancer patients who underwent a combination therapy of VMAT and LDR with hydrogel spacer insertion

**DOI:** 10.1002/acm2.13584

**Published:** 2022-03-14

**Authors:** Honglai Zhang, Lin Wang, Adam C. Riegel, Jeffrey Antone, Louis Potters, Lucille Lee, Yijian Cao

**Affiliations:** ^1^ Department of Radiation Medicine Northwell Health Cancer Institute, Lake Success New York USA; ^2^ Department of Radiation Medicine Zucker School of Medicine at Northwell/Hofstra Hempstead New York USA

**Keywords:** biological effective dose (BED), hydrogel spacer, LDR brachytherapy, prostate cancer, VMAT and PSI

## Abstract

This study aimed to evaluate rectal dose reduction in prostate cancer patients who underwent a combination of volumetric modulated arc therapy (VMAT) and low‐dose‐rate (LDR) brachytherapy with insertion of hydrogel spacer (SpaceOAR). For this study, 35 patients receiving hydrogel spacer and 30 patients receiving no spacer were retrospectively enrolled. Patient was treated to doses of 45 Gy to the primary tumor site and nodal regions over 25 fractions using VMAT and 100 Gy to the prostate using prostate seed implant (PSI). In VMAT plans of patients with no spacer, mean doses of rectal wall were 43.6, 42.4, 40.1, and 28.8 Gy to the volume of 0.5, 1, 2, and 5 cm^3^, respectively. In patients with SpaceOAR, average rectal wall doses decreased to 39.0, 36.9, 33.5, and 23.9 Gy to the volume of 0.5, 1, 2, and 5 cm^3^, respectively (*p* < 0.01). In PSI plans, rectal wall doses were on average 78.5, 60.9, 41.8, and 14.8 Gy to the volume of 0.5, 1, 2, and 5 cm^3^, respectively, in patients without spacer. In contrast, the doses decreased to 34.5, 28.4, 20.6 (*p* < 0.01), and 8.5 Gy (*p* < 0.05) to rectal wall volume of 0.5, 1, 2, and 5 cm^3^, respectively, in patient with SpaceOAR. To demonstrate rectal sum dose sparing, dose‐biological effective dose (BED) calculation was accomplished in those patients who showed >60% overlap of rectal volumetric doses between VMAT and PSI. In patients with SpaceOAR, average BED_sum_ was decreased up to 34%, which was 90.1, 78.9, 65.9, and 40.8 Gy to rectal volume of 0.5, 1, 2, and 5 cm^3^, respectively, in comparison to 137.4, 116.7, 93.0, and 50.2 Gy to the volume of 0.5, 1, 2, and 5 cm^3^, respectively, in those with no spacer. Our result suggested a significant reduction of rectal doses in those patients who underwent a combination of VMAT and LDR with hydrogel spacer placement.

## 1 INTRODUCTION

Radiotherapy is the main nonsurgical treatment for patients with prostate cancer. Radiation dose escalation results in improved clinical outcomes, but it also increases risk of rectal toxicity.^1^, ^2^, ^3^ Rectal toxicity is dependent on rectal dose, which is ultimately associated with distance from the prostate. One approach in reducing rectal dose is to create an interspace between prostate and rectum, which can be achieved by insertion of polyethylene glycol (PEG) hydrogel.^3^ Injection of absorbable PEG hydrogel in creation of peri‐rectal spacer is a highly successful technique to generate the distance of approximately 1–1.5 cm between the prostate and rectum.^4^ Shape of hydrogel spacer can be maintained for 3 months during radiation treatment.^5^, ^6^, ^7^

Several studies have shown significant dosimetric impact by insertion of hydrogel spacer on rectum, and consequently, lower risk of rectal toxicity was observed during and after radiation treatment.^8^, ^9^ However, those data focused on assessment of rectal dose and its toxicity in patients undergoing either external beam radiation therapy (EBRT)^10^, ^11^, ^12^, ^13^, ^14^, ^15^ or brachytherapy.^5^, ^16^, ^17^, ^18^ There are limited data in the literature looking at rectal dose sparing in patients undergoing a combination of EBRT and low dose‐rate (LDR) brachytherapy with hydrogel spacer. The combined modality treatment allows for the delivery of an escalated biologically effective dose, which improves local disease control and distant metastasis‐free survival in prostate cancer patients.^19^ In this retrospective study, biological effective dose (BED) was employed in comparison of rectal doses in prostate cancer patients who did and did not have rectal spacer placed, providing a means in estimation of dosimetric impact by hydrogel spacer insertion on rectum in prostate cancer patients who underwent LDR after EBRT.

## 2 MATERIALS AND METHODS

### 2.1 Selection of the patients

In this retrospective study, 65 patients with prostate cancer including 35 patients receiving hydrogel spacer (SpaceOAR) and 30 patients receiving no spacer were enrolled. Selection of the patients was based on the following criteria: (1) patient was treated to doses of 45 Gy over 25 fractions using volumetric modulated arc therapy (VMAT) and a boost 100 Gy using Palladium‐103 (Pd‐103) seed implant; (2) prostate plus lymph nodes in pelvic regions were included in the treatment.

### 2.2 Insertion of hydrogel spacer

Insertion of hydrogel spacer has been described in detail by other studies.^10^, ^20^ Briefly, patients received a transrectal ultrasound‐guided transperineal injection of 10 ml PEG hydrogels (SpaceOAR System, Augmenix Waltham, MA). A needle was advanced into the retroprostatic space below the Denonvillier's fascial and above the anterior rectal wall using the sagittal plane of the transrectal ultrasound. The hydrogel spacer was formed by injecting two separate liquids that solidified into a gel within 7–10 s of injection.

### 2.3 Patient simulation and contour

Non‐contrast computed tomography (CT) simulation was performed at 0.3 cm intervals as per our standard procedure, approximately 2 weeks after placement of SpaceOAR. Co‐registration of the CT and magnetic resonance imaging (MRI) was performed for verification of the spacer, delineation of target and normal tissue structures using Velocity (Version 4.0, Varian Medical System Inc., Atlanta, GA). Target volumes of the prostate and high‐risk nodal region were outlined, known as PTV_45_ according to the prescriptions. Normal tissue structures, including rectum, bladder, small bowel, large bowel, and femur heads, were contoured as organ‐at‐risk (OAR). Rectum was outlined as a solid organ, and rectal wall was defined as 0.4 cm thickness inside the outer contour of rectum. All contours underwent departmental peer review prior to planning in Eclipse (Version 15.6, Varian Medical System Inc., Palo Alto, CA).

### 2.4 Plan dosimetry and statistical analysis

The EBRT portion of therapy was delivered using conventional fractionation (45 Gy in 25 fractions). Two‐arc VMAT plan was generated using 6MV photons. The anisotropic analytical algorithm (AAA) was used as dose calculation model with a grid size of 0.25 × 0.25 × 0.25 cm^3^ and heterogeneity correction applied. Two weeks after VMAT, patient underwent LDR brachytherapy that was delivered using Pd‐103 seed implant.^21^, ^22^, ^23^, ^24^ Intraoperative planning was performed using VariSeed (Version 9.0, Varian Medical System Inc., Palo Alto, CA). Target dose coverage and OAR dose constraints were determined following departmental treatment directives and RTOG0924 guidelines.^25^ CT‐based dosimetry of post‐prostate seed implant (PSI) was completed 3–4 weeks after the intraoperative procedure.

Both VMAT and PSI plans were exported in DICOM‐RT format to Velocity for examination of target dose coverage and OAR dose sparing. Rectal wall dose was evaluated using the VMAT structure set after completing both rigid and deformable image registration between PSI image and VMAT image datasets. Briefly, the CT images for PSI plan were aligned with the images for VMAT plan by using mutual information‐based rigid registration, and the rectal volumetric dose was examined. To further limit the impact of rectal wall positioning uncertainties between images acquired on different days, B‐spline deformable image registration was applied in each patient. In Velocity, a region of interest was generated by expanding the PTV and rectum by 1 cm in three dimensions. Structure‐based deformable registration was performed using the 1 cm expanded volume around the PTV and rectum. Percent difference of the rectal doses evaluated under two types of image registration was presented in Table 2. Volumetric doses of rectal wall, including volume of 0.5, 1, 2, and 5 cm^3^, were denoted as *D*_0.5cc_, *D*_1cc_, *D*_2cc_, and *D*_5cc_. Dose coverage of PTV_45_ was showed in maximum pixel dose (*D*_max_), relative dose *D*_95_, and conformity index (CI) in VMAT plan. CI was defined as percent volume of target PTV covered by 100% of the prescribed dose. Dose CI above 70% had been previously considered acceptable in the literature.^26^ In PSI plan, target coverage was examined in relative dose *D*_90_, *V*_100_, and *V*_150_. Statistical significance of dosimetric outcomes between two‐group patients was analyzed using an unpaired Student's *t‐*test (Microsoft Excel 365).

### 2.5 Measurement of volumetric dose overlap

Percent overlap of the rectal volumetric doses was examined between VMAT and PSI plans in each patient in Velocity. Briefly, each volumetric dose, *i.e*. *D*_0.5cc_, *D*_1cc_, *D*_2cc_, and *D*_5cc_, was converted to a structure, and then intersection of the dose structures was measured in absolute volume (cm^3^). Using Velocity, we were able to analyze percent overlap in each pair of the volumetric doses obtained from VMAT and PSI in each patient. Although deformable image registration reduced some alignment uncertainty in the rectal wall, complete overlap of the volumetric doses was unlikely to occur, especially in the small volume dose, *i.e*. *D*_0.5cc_. To obtain rectal sum dose, the patient who had greater than 60% overlapping in *D*_0.5cc_, *D*_1cc_, *D*_2cc_, and D_5cc_ would be favored in selection in BED_sum_ calculation.

### 2.6 Biological effective dose calculation

The linear‐quadratic model has been widely accepted in describing cell response to radiation.^27^, ^28^, ^29^, ^30^ Rectal dose‐BED conversion in VMAT plan over *n* treatment fractions, dose *d* per fraction was completed by using Equation (1):

(1) $$\mathrm{BED}\;=\;nd\left(1+\frac{d}{\alpha /\beta }\right)$$

where *α* and *β* were the radiosensitivity parameters for the linear‐quadratic responses, respectively. The quotient *α*/*β* was the dose at which linear and quadratic terms contribute equally to biological response. Cell proliferation for rectum, as a normal tissue, was not taken into account in treatment period of prostate cancer patient.

For rectal dose‐BED conversion in PSI plan, the equivalent BED calculation, derived by Zhang ^31^ and Dale,^32^ was formulated as shown in Equation (2):

(2) $$\mathrm{BED}\;=\;D\;\left[1+\;\frac{D_{0}}{\left(\mu +\lambda \right)\left(\alpha /\beta \right)}\right]$$

where *D* was the dose delivered to the full decay, D_0_ = D * λ was defined as initial dose rate, *λ* was decay constant, and *μ* was the sublethal repair coefficient. Equation (2) assumed no significant repopulation of rectum over treatment course and sublethal repair half‐time (*T_r_*) was significantly longer for late responding normal tissues. In this study, dose‐BED conversion was completed for rectal wall doses using the parameters according to AAPM report TG‐137,^33^ and Pritz's study,^34^ and Guerrero's report.^35^ Briefly, *D* was the rectal dose delivered to the full decay (*D*_0.5cc_, *D*_1cc_, *D*_2cc_, and *D*_5cc_). *λ* was Pd‐103 decay constant (*λ*_ _= 0.0408 day^−1^). The rectal repair coefficient *μ* was 43.3217day^−1^ (*μ* = ln2/T_r_), where *T_r_* was sublethal repair half time (*T_r_ *= 0.016 day). Rectal *α/β* ratio was 4 Gy (*α* = 0.048 Gy^−1^; *β* = 0.012 Gy^−2^).

## 3 RESULTS

### 3.1 Evaluation of target dose coverage

The target dose coverage in both VMAT and PSI plans were evaluated in each patient and did not appear to be significantly affected by the use of a hydrogel spacer (Table 1). In the VMAT plan, PTV_45_ coverage was examined in relative dose *D*_95_, maximum pixel doses (*D*_max_), and CI. Dose *D*_95_ on each PTV_45_ was adequate to satisfy *D*_95_ > 42.8 Gy that was 95% of the prescription, as required. The average *D*_95_ was 44.3 Gy in patients with SpaceOAR and 44.2 Gy in those without spacer. Maximum pixel dose was an average of 48.3 and 48.5 Gy in patients having SpacerOAR and those having no spacer, respectively, which satisfied the maximum dose less than 110% prescription dose objective. The average CI for PTV_45_ was 92.1% in patients with SpaceOAR and 91.8% in those without spacer. A significant difference of PTV_45_ coverage was not observed between the two groups of patients (*p* > 0.05). In the PSI plan, target coverage was assessed with *D*_90_, *V*_100_, and *V*_150_. The average *D*_90_ was 100.9 and 103.5 Gy in patients with and without the insertion, respectively. Average *V*_150_ was 57.8% in patients with SpaceOAR and 63.8% in patients with no spacer. On average, *V*_100_ was found to be 88.9% in patient having SpaceOAR and 91.8% in those having no spacer. There was no statistically significant difference in *D*_90_, *V*_100_, and *V*_150_ found between the two groups (*p* > 0.05).

**TABLE 1:** Target dose coverage

PTV_45_ coverage in VMAT plan			Prostate coverage in PSI plan		
	SpaceOAR	No spacer		SpaceOAR	No spacer
*D*_95_ ± SD, Gy	44.3 ± 0.5	44.2 ± 0.8	*D*_90_ ± SD, Gy	100.9 ± 10.8	103.5 ± 19.4
*D*_max_± SD, Gy	48.3 ± 0.5	48.5 ± 0.7	*V*_150_ ± SD, %	57.8 ± 9.2	63.8 ± 9.5
CI ± SD, %	92.1 ± 7.4	91.8 ± 13.5	*V*_100_ ± SD, %	88.9 ± 5.7	91.8 ± 4.5

Abbreviations: CI, conformity index; PSI, prostate seed implant; SD, standard deviation; VMAT, volumetric modulated arc therapy.

### 3.2 Examination of rectal wall dose

**FIGURE 1:**
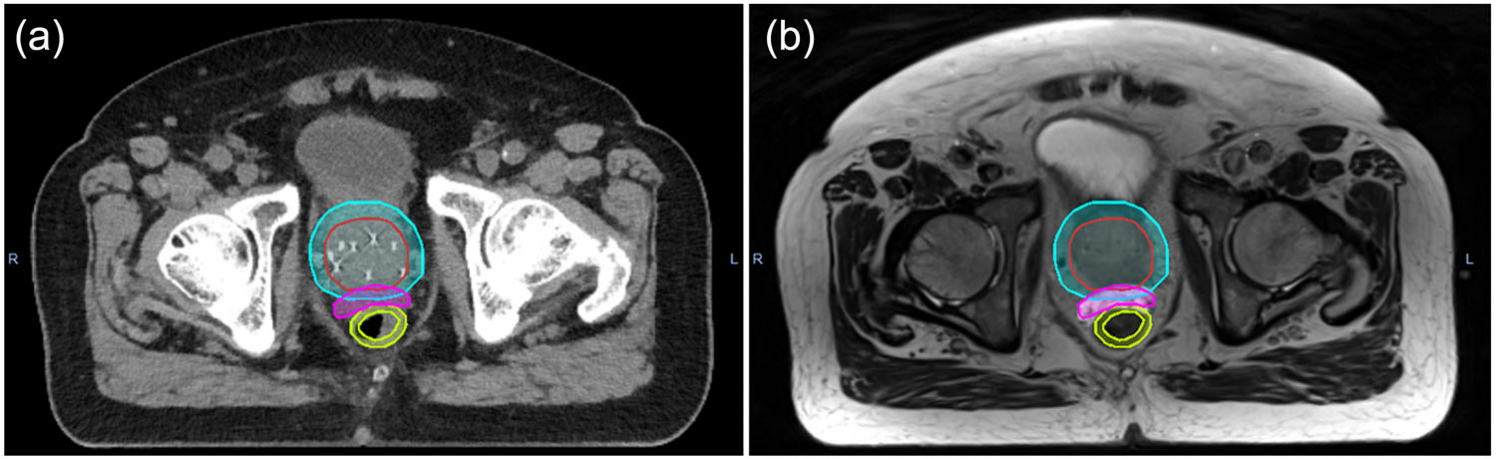
An example of computed tomography (CT) (a) and magnetic resonance imaging (MRI) (b). The hydrogel spacer (pink) was inserted between prostate (red) and rectal wall (light green). PTV_45_ (green) was prostate (red) with expansion of the margins.

An interspace created by insertion of hydrogel spacer was outlined as SpaceOAR (Figure 1). An average volume was 8.8 (± 2.4) cm^3^ and an average distance between prostate and rectum was about 1 (± 0.2) cm. An average volume of rectal wall was 13.1 cm^3^ with range from 8.8 to 21.2 cm^3^ in patients with SpaceOAR, and 12.6 cm^3^ in range of 8.7–17.2 cm^3^ in those with no spacer. There was no significant difference observed between the two groups.

Combined volumetric doses of the rectal wall (*D*_0.5cc_, *D*_1cc_, *D*_2cc_, and D_5cc_) were evaluated in the VMAT structural set in Velocity. Percent difference in the rectal wall doses measured under rigid and deformable image registrations is depicted in Table 2, showing that less than 1% dose difference was found in 24 of 35 patients with spacer and 18 of 30 patients without spacer. The doses varied in 1%‐5% and 5%‐10% were also seen in the patients. Furthermore, greater than 10% difference of the dose measurement was observed in two patients with spacer and five patients without spacer. In agreement with the study by Oh and Kim,^36^ which suggested that deformable image registration can bring an opportunity of response evaluation and cumulative dose estimation, we focused on rectal wall doses measured under deformable registration.

**TABLE 2:** Percent difference of rectal wall doses between rigid and deformable image registration

	# Patients	
Rectal wall doses % difference	SpaceOAR	No spacer
<1%	24	18
1–5%	5	4
5–10%	4	3
>10%	2	5

Rectal wall doses were significantly decreased in both VMAT (Table 3) and PSI plans (Table 4) in patients receiving SpaceOAR in comparison with those having no spacer. In VMAT plans of patients without spacer insertion, rectal wall doses were on average of 43.6, 42.4, 40.1, and 28.8 Gy to the volume of 0.5, 1, 2, and 5 cm^3^, respectively. In patients with SpaceOAR, rectal wall doses were reduced significantly (*p *< 0.01), on average of 39.0, 36.9, 33.5, and 23.9 Gy to rectal volume of 0.5, 1, 2, and 5 cm^3^, respectively. In PSI plans, on average, rectal wall doses in patients without the spacer were 78.5, 60.9, 41.8, and 14.8 Gy to the volume of 0.5, 1, 2, and 5 cm^3^, respectively. Significant decreases of the doses were observed in patients with SpaceOAR, which were on average 34.5, 28.4, 20.6 (*p *< 0.01), and 8.5 Gy (*p* < 0.05) to rectal wall volume of 0.5, 1, 2, and 5 cm^3^, respectively.

**TABLE 3:** Rectal wall dose in volumetric modulated arc therapy (VMAT) plan

	*n*	*D*_0.5cc_	*D*_1cc_	*D*_2cc_	*D*_5cc_
SpaceOAR (X ± SD, Gy)	35	39.0 ± 5.1	36.9 ± 5.6	33.5 ± 5.8	23.9 ± 5.5
No spacer (X ± SD, Gy)	30	43.6 ± 2.7	42.4 ± 3.4	40.1 ± 4.3	28.8 ± 6.4
*t‐*test		*p *< 0.01	*p *< 0.01	*p *< 0.01	*p *< 0.01

**TABLE 4:** Rectal wall dose in prostate seed implant (PSI) plan

	*n*	*D*_0.5cc_	*D*_1cc_	*D*_2cc_	*D*_5cc_
SpaceOAR (X ± SD, Gy)	35	34.5 ± 15.4	28.4 ± 12.5	20.6 ± 8.8	8.5 ± 3.4
No spacer (X ± SD, Gy)	30	78.5 ± 48.3	60.9 ± 37.3	41.8 ± 27.9	14.8 ± 13.7
*t‐*test		*p *< 0.01	*p *< 0.01	*p *< 0.01	*p *< 0.05

### 3.3 Calculation of biological effective dose

**FIGURE 2:**
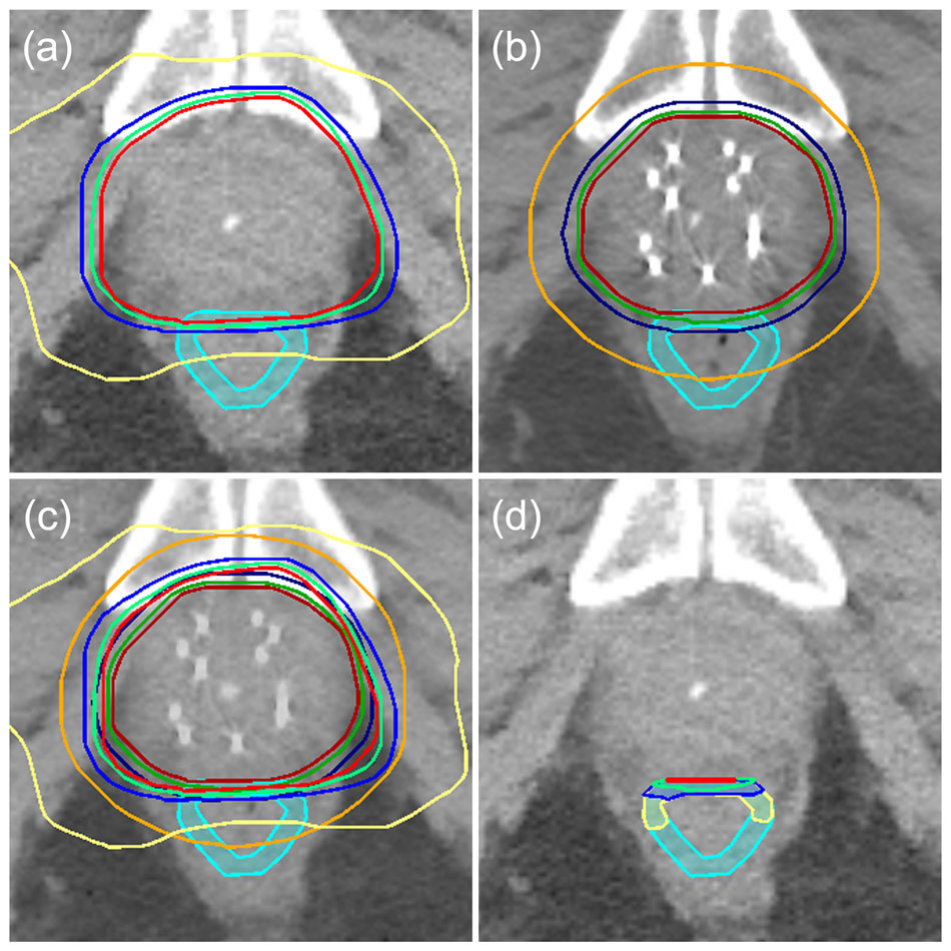
An example of computed tpmpgrapy (CT) image depicts isodose lines in volumetric modulated arc therapy (VMAT) and prostate seed implant (PSI). (a) In VMAT plan, volumetric dose *D*_0.5cc_, *D*_1cc_, *D*_2cc_, and *D*_5cc_ on rectal wall was donated by isodose 40.4 (red), 38.2 (green), 34.7 (blue), and 23.6 Gy (yellow), respectively. (b) In PSI plan, volumetric dose *D*_0.5cc_, *D*_1cc_, *D*_2cc_, and *D*_5cc_ on rectal wall was contributed by isodose 45.1 (dark red), 38.3 (dark green), 28.6 (dark blue), and 10.8 Gy (orange), respectively. (c) Merge of a and b. (d) Intersections of each pair of the isodoses on rectal wall between two plans, showing the overlap volume of *D*_0.5cc_ (red), *D*_1cc_ (green), *D*_2cc_ (blue), and *D*_5cc_ (yellow). Contour of rectal wall is in cyan.

**FIGURE 3:**
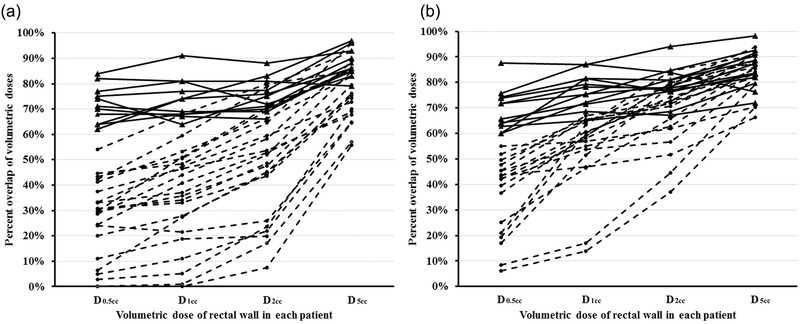
Percent overlap of the volumetric doses of rectal wall between volumetric modulated arc therapy (VMAT) and prostate seed implant (PSI) in each patient. More than 60% overlaps in the examined doses were observed in 11 patients with SpaceOAR (a, solid line) and 12 patients with no spacer (b, solid line). Rectal dose‐BED conversion was completed in those patients. Patients showing less than 60% overlaps in the dose *D*_0.5cc_ were excluded in selection for biological effective dose (BED) calculation (a, b; dash line).

The rectal dose‐BED conversion was carried out in each patient. To further combine the BED to sum dose (BED_sum_), it was necessary to examine whether the volumetric doses overlapped in VMAT and PSI. Using Velocity, we calculated percent intersection of the dose volumes in VMAT and PSI plans (Figures 2 and 3). In general, larger volume dose yielded greater overlap. For example, *D*_5cc_ demonstrated that more than 90% overlap occurred in five patients and 60% in all patients, except for two patients in the spacer group, and more than 90% overlap in eight patients and 60% in all patients in no spacer group. However, relatively high percent overlap was unlikely observed in smaller volumetric doses, especially in dose *D*_0.5cc_, which might result from anatomical feature of rectum plus physical characterization of radiation distribution. In consideration of more than 0.3 cm^3^ volume overlapping in 0.5 cm^3^ dose volume (*D*_0.5cc_) between VMAT and PSI in individual patient, for example, we proposed that 60% or more overlapping would be sufficient in analysis of rectal sum doses (BED_sum_). There were 11 of 35 patients with SpaceOAR and 12 of 30 patients with no spacer showing greater than 60% overlap in all examined doses, who were selected in BED_sum_ calculation.

In those 12 patients with no spacer, average BED_sum_ was 137.4, 116.7, 93.0, and 50.2 Gy to rectal wall dose volume of 0.5, 1, 2, and 5 cm^3^, respectively (Table 5). Average BED_sum_ was decreased to 90.1, 78.9, 65.9, and 40.8 Gy to rectal volume of 0.5, 1, 2, and 5 cm^3^, respectively, in the 11 patients with SpaceOAR (Table 5). The reductions were 34%, 32%, and 30% to BED_sum_0.5cc_, BED_sum_1cc_, and BED_sum_2cc_, respectively (*p* < 0.01). BED_sum_5cc_ decreased by 18%, but it was still not statistically significant (*p* = 0.051), possibly due to the limited sample size.

**TABLE 5:** BED_sum_ of rectal wall dose

	*N*	BED_sum_0.5cc_	BED_sum_1cc_	BED_sum_2cc_	BED_sum_5cc_
SpaceOAR (X ± SD, Gy)	11	90.1 ± 20.1	78.9 ± 17.1	65.9 ± 13.8	40.8 ± 9.3
No spacer (X ± SD, Gy)	12	137.4 ± 38.1	116.7 ± 28.1	93.0 ± 18.5	50.2 ± 12.1
*t‐*test		*p *< 0.01	*p *< 0.01	*p *< 0.01	*p *> 0.05

## 4 DISCUSSION

This study assessed dosimetric impact by hydrogel spacer insertion on rectal sum dose using BED calculation in prostate cancer patients who underwent EBRT and LDR combination therapy. It has been well documented that late rectal toxicity is correlated to the volume of the anterior rectal wall that receives the highest radiation dose.^11^, ^37^, ^38^, ^39^ The use of rectal hydrogel spacer has been shown to be efficacious in reducing the rectal dose.^40^, ^41^, ^42^ A recent report by Nehlsen et al. shows a 47% reduction of the rectal volume receiving 100 Gy in patients receiving LDR after EBRT with hydrogel spacer.^43^ In this study, we focused on analysis of the rectal sum dose (BED_sum_) in patients who underwent a combination of EBRT and LDR therapy with hydrogel spacer, and showed decreases of rectal BED_sum_0.5cc_, BED_sum_1cc_, and BED_sum_2cc_ by up to 34% in comparison with those patients who did not have spacer placement.

The current work has a few limitations in demonstration of rectal BED_sum_. First, due to changes in bladder and rectal filling, changes in the position of the anterior rectal wall were inevitable. This led to uncertainties in rectal dose volume, that is, *D*_0.5cc_, *D*_1cc_, *D*_2cc_, and *D*_5cc_, between VMAT and PSI CT image sets that were acquired on different days. As this could impact rectal BED summation, we used deformable image registration to mitigate this concern. Certainly, the higher percentage of the volumetric dose intersection between VMAT and PSI was seen, the more reasonable estimation of rectal sum dose would be. We showed that greater than 90% overlap could be seen in relatively large volumetric doses, such as *D*_5cc_, in both groups of patients, but 100% overlap was unlikely. Meanwhile, it was feasible to see greater than 60% overlap in the volumetric doses *D*_0.5cc_ in some patients. Determining the overlap threshold for proper dose summation could be challenged. Here, we proposed that intersection volume 0.3 cm^3^ or more occurred in the examined dose volume 0.5 cm^3^ (*D*_0.5cc_), *i.e*. 60% overlapping, would be considered a substantially impressive volume in affecting rectal BED_sum_0.5cc_. Therefore, rectal BED calculation was carried out in those patients who showed 60% or more overlaps of the volumetric doses between VMAT and PSI. We believe that the BED_sum_ could be reasonably useful in estimating rectal sum dose, even in the condition of 100% dose intersection between VMAT and PSI, as the worst‐case scenario. Furthermore, image registration could be another limitation affecting measurement of the dose overlapping. After considering rigid and deformable image registration in Velocity, we compared the rectal doses in each patient using both techniques and decided to utilize deformable image registration because it has been reported that deformable image registration can be used for response evaluation and cumulative dose estimation.^36^ A study has also demonstrated that reasonable accuracy could be achieved using B‐spline model of deformable image registration in Velocity.^44^ In our institute, a prospective study has been launched in evaluation of rectal dose sparing using BED_sum_ calculation and correlation with rectal toxicity during and after the treatment, an attempt to develop planning guidelines that incorporate summed dosimetry. Furthermore, a novel method using voxel‐by‐voxel dose summation to improve the accuracy of combined modality dosimetry has been developed in our institute^45^ and is being considered for application in our prospective study. Combined dose BED_sum_ could potentially yield new planning guidelines.

## 5 CONCLUSION

We concluded that rectal BED_sum_ calculation would provide valuable information in assessment of dosimetric impact by insertion of hydrogel spacer on rectal sum dose sparing in prostate cancer patients who underwent a combination of EBRT and LDR therapy. Statistically significant dosimetric advantages in rectal wall sparing were observed in favor of patients with hydrogel spacer, indicating up to 34% reduction of rectal wall volumetric dose compared with patients treated without spacer. This trend held for combined dosimetry as well as EBRT and LDR components.

## CONFLICT OF INTEREST

The authors declare no conflict of interest for this work.

## AUTHOR CONTRIBUTIONS

Project design, data collection, EBRT planning, data analysis, and manuscript writing: Honglai Zhang. Performing prostate implant and treatment planning, post‐implant dosimetry, and data collection: Lin Wang. Performing prostate implant and treatment planning, post‐implant dosimetry, data analysis, and manuscript revision: Adam C. Riegel. EBRT treatment planning: Jeffrey Antone. Project development, performing prostate implant, and manuscript revision: Louis Potters. Project development, performing prostate implant, and manuscript revision: Lucille Lee. Project design, data analysis, and manuscript writing: Yijian Cao
